# A risk model of 10 aging‐related genes for predicting survival and immune response in triple‐negative breast cancer

**DOI:** 10.1002/cam4.4674

**Published:** 2022-03-16

**Authors:** Xia Yang, Yanhua Sun, Xia Liu, Zhinong Jiang

**Affiliations:** ^1^ Department of pathology Sir Run Run Shaw hospital of Zhejiang University School of Medicine Hangzhou China; ^2^ Department of Pathology first affiliated hospital of Shenzhen University Shenzhen China

**Keywords:** aging‐related genes, immune response, prognosis, triple‐negative breast cancer

## Abstract

Accumulated studies showed that the clinical significance of aging on the development and malignancy of tumors, while the relationship between aging and the prognosis, immune response in triple‐negative breast cancer (TNBC) has not been well clarified. Here, we constructed a risk model of 10 prognostic aging‐related genes (ARGs) from METABRIC database. Then, TNBC patients were classified into high‐ and low‐risk groups, the survival diversity, immune response, genomic function, and tumor mutation burden (TMB) between different risk groups were explored in METABRIC, TCGA, and GSE58812 cohorts. Results showed that patients in the high‐risk group had poorer survival outcomes compared to their counterparts (all *p* < 0.05), and the nomogram we established showed reliable prediction ability for survival in TNBC patients. Besides, TNBC patients with high‐risk scores had a lower expression of immune checkpoint markers and a lower fraction of activated immune cells. Furthermore, GSEA showed that Notch signaling pathway was significantly enriched in the high‐risk group. Thus, a risk model based on the aging‐related genes was developed and validated in this study, which may serve as a potential biomarker for prognosis and personalized treatment in TNBCs.

## Graphical abstract

Using the mRNA profiles, clinical features and mutation data of TNBC from METABRIC, TCGA and GSE58812 databases, we identified a risk signature of 10 prognostic aging‐related genes (ARGs) to explore the survival diversity, immune status, genomic function, tumor mutation burden (TMB) and therapeutic response between different risk groups, which possibly have potential function to the optimization of risk stratification for prognosis and personalized treatment in TNBC patients.

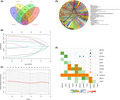

## 1 INTRODUCTION

Triple‐negative breast cancer (TNBC) is a special subtype of breast cancer that lacks the expression of ER, PR, and HER2.^1^ Universal treatment strategies have been introduced for TNBC in the last decades, but the clinical outcomes of TNBC remain poor due to its malignant biological behavior and lack of targeted therapeutic methods.^2^, ^3^, ^4^, ^5^ Thus, comprehensive studies are warranted to understand the biological profile and mechanism of TNBC for developing novel effective therapeutic strategies.

Cell senescence is a universal biological feature of organisms, which occurs in response to exogenous and endogenous stresses.^6^, ^7^ Generally, senescent cells have the following characteristics: cell cycle arrest in G1 and resistance to apoptosis; retain some basic metabolic activities and senescence‐associated secretory phenotype (SASP).^8^, ^9^ Cell cycle arrest of senescent cells is regulated by the p53/p21^CIP1^ and p16^INK4a^/Rb tumor suppressor pathway.^10^, ^11^ Unlike quiescent cells, senescent cells could not respond to mitosis or growth factor stimulation, they are unable to re‐enter the cell cycle even under favorable growth conditions. SASP, which includes the secretion of numerous proinflammatory cytokines, chemokines, extracellular matrix proteases, and growth factors, plays a crucial role in aging‐related disease.^9^, ^12^

Currently, great attention has been paid to the association between aging and cancer. At the first glance, they seem to be opposite processes: cancer is the result of an abnormal increase in cellular adaptation, while aging is characterized by a loss of cellular adaptation. Further exploring showed that tumor and aging have the common feature: the biological basis of cancer cells, namely abnormal mitochondrial function, is consistent with the decline of mitochondrial function in the aging process.^13^, ^14^ Furthermore, senescent cells promote tumor progression by influencing tumor microenvironment and inducing chromosomal instability.^15^, ^16^ Thus, cancer and aging can be thought of two different manifestations of the same underlying process.

Previous studies have confirmed that aging is closely related to the occurrence and development of tumors. Senescent cells can trigger inflammatory response and involvement in the underlying mechanism of tumor progression.^13^, ^14^, ^17^, ^18^, ^19^ On the one hand, senescent cells interact with immune cells by secreting a variety of cytokines and chemokines, thus further removing senescent tumor cells and inhibiting tumor development.^20^, ^21^ On the other hand, some inflammatory factors in SASP may escape immune surveillance and promote tumor malignancy by paracrine pathway.^22^, ^23^ Furthermore, senescence‐related inflammation response (SIR), a low‐grade atypical inflammatory response in senescent epithelial cells, can promote the tumorigenesis and development of tumors by secreting a variety of pro‐cancer inflammatory factors.^24^, ^25^

Conventional wisdom holds that TNBC is more likely to occur in younger patients. The relationship between aging and tumor initiation and progression in this cancer type has not been well clarified. An increasing number of researches hold that aging may involve the induction and malignancy of TNBC by complex underlying mechanisms. First, the cumulative mutation events caused by aging partially explain the relationship between aging and this heterogeneous cancer type.^26^ For instance, the frequency of diver mutations in genes such as BRCA1/2 or TP53 is higher in TNBC than other breast cancer subtypes, which also involve in the regulation of cell senescence.^27^, ^28^ Besides, DNA damage gene therapy (mainly radiotherapy and chemotherapy), the main treatment for TNBC patients in clinical practice, can induce senescent tumor cells to secrete SASP, then promote tumor migration and metastasis. Previous research found that chemotherapy‐resistant TNBC cells occurred cell senescence, then maintain a dormant status, which would become the root of disease recurrence and metastases.^29^, ^30^ Thus, elaborating the aging status in TNBC may contribute to understand the biological profile and mechanism of TNBC.

The human aging genome resource (HAGR) is a database designed to help researchers explore the characteristics of human aging genes.^31^ To evaluate the underlying biological functions of aging‐related genes (ARGs) in TNBC, we initially downloaded ARGs from HAGR dataset. Then, the gene expression profiles of TNBC patients from METABRIC, TCGA and GSE58812 datasets were obtained to establish and substantiate an aging‐related gene signature for predicting survival, immune response, and therapeutic response in TNBC by performing a comprehensive bioinformatics analysis.

## 2 MATERIALS AND METHODS

### 2.1 Data acquisition

The mRNA expression profiles of TNBC patients with clinical and survival information were acquired from the Molecular Taxonomy of Breast Cancer International Consortium (METABRIC, *N* = 221) (http://www.cbioportal.org/), the cancer genome atlas (TCGA, *N* = 142) (https://portal.gdc.cancer.gov/), and GSE58812 (*N* = 107) (http://www.ncbi.nlm.nih.gov/geo/). A total of 307 ARGs were collected from the human aging genome resource (HAGR) dataset (http://genomics.senescence.info/genes/). The inclusion criteria were listed as follows: female, diagnosed TNBC, the survival time more than 30 days. The METABRIC dataset served as a training set to construct a risk model based on the selected ARGs, the TCGA and GSE58812 datasets acted as testing sets to validate the established model.

### 2.2 Identify prognostic ARGs and establish a risk model based on selected ARGs

Using the R package “survival”, prognosis‐associated ARGs were selected according to the standard of *p* < 0.05 by univariate Cox analysis. Then, using the R package “glmnet”,^32^ the most robust prognostic ARGs were selected in the LASSO Cox regression. Finally, a risk model based on 10 selected ARGs was established.

The calculated formula of risk score as follows:

$$\text{Risk score}=\sum i\;\text{Coefficient}\left(\mathrm{ARG}\right)\times \text{Expression}\left(\mathrm{ARG}\right).$$

Where coefficient (ARG) and expression (ARG) present the corresponding coefficient and the expression level of each selected prognostic ARG, respectively. Then, patients were divided into low‐ and high‐risk groups according to the median risk score.

### 2.3 Evaluation of the immune landscape

The compositional fraction of 22 immunocytes of each sample was calculated applying the CIBERSORT algorithm.^33^ The immune‐related efficiency of each sample was estimated through the “MCPcounter” package.^34^ Immune and stromal scores of TNBC patients were evaluated using the “estimate” package.^35^ Besides, the expression of key immune profiles and proinflammatory factors between the low‐ and high‐risk groups were compared using the Wilcoxon test.

### 2.4 Overall genes mutation and tumor mutation burden analysis

The TNBC mutational data were obtained from the TCGA dataset. The diversity of TMB scores between the low‐ and high‐risk groups was calculated. Then, the overall genes mutation were estimated in different risk groups using the R package maftools.^36^

### 2.5 Exploration of potential compounds targeting the selected ARGS

To explore potential compounds targeting the ARGs‐based risk model for the treatment of TNBC, we calculated the therapeutic response based on the IC50 value of various molecular obtained from the CellMiner database for each sample.^37^

### 2.6 Functional analysis

Using the Clusterprofile package,^38^ GSEA was performed to explore the potential differences of GO and KEGG pathway between the low‐ and high‐risk groups. The FDR < 0.25 and *p* < 0.05 were considered significantly enriched.

### 2.7 Establishment and Validation of a prognostic nomogram

Using the R package “rms”,^39^ a nomogram was constructed based on the independent prognostic factors (age, tumor stage, node stage, and the risk model) in METABRIC cohort. The predictive accuracy of the nomogram was estimated by calibration plots and time‐ROC analysis in both the training and validation sets.

### 2.8 Validation of the bioinformatics results using RT‐qPCR assay

64 paired TNBC tissues (T) and adjacent normal tissues (N) were collected from the First Affiliated Hospital of Shenzhen University from May 2016 to June 2020. The median age of these TNBC patients was 56.8, 36.0% (23/64), 54.7% (35/64), and 9.3% (6/64) of patients had tumor size as ≤2 cm, 2‐5 cm, and >5 cm, respectively. Furthermore, 23.4% (15/64) of patients had positive node involvement and 14.0% (9/64) had distant **metastasis.** Total RNA was extracted using TRIzol reagent (Invitrogen, USA) according to the manufacturer's protocol. Reverse transcription was conducted using PrimeScript RT MasterMix (Takara, China) to synthesize cDNA. RT‐qPCR was performed using SYBR Green PCR MasterMix (Takara, China) according to the manufacturer's protocol. The RT‐qPCR primers were designed by Primer Bank (https://pga.mgh.harvard.edu/primerbank/) and were listed in (Table S1), Data were analyzed using 2‐ΔΔCt method and the experiment was repeated three times in total, Target genes mRNA expression were normalized to those of GAPDH.

## 3 RESULTS

### 3.1 Selection of prognostic ARGs and construction of a risk model

**FIGURE 1:**
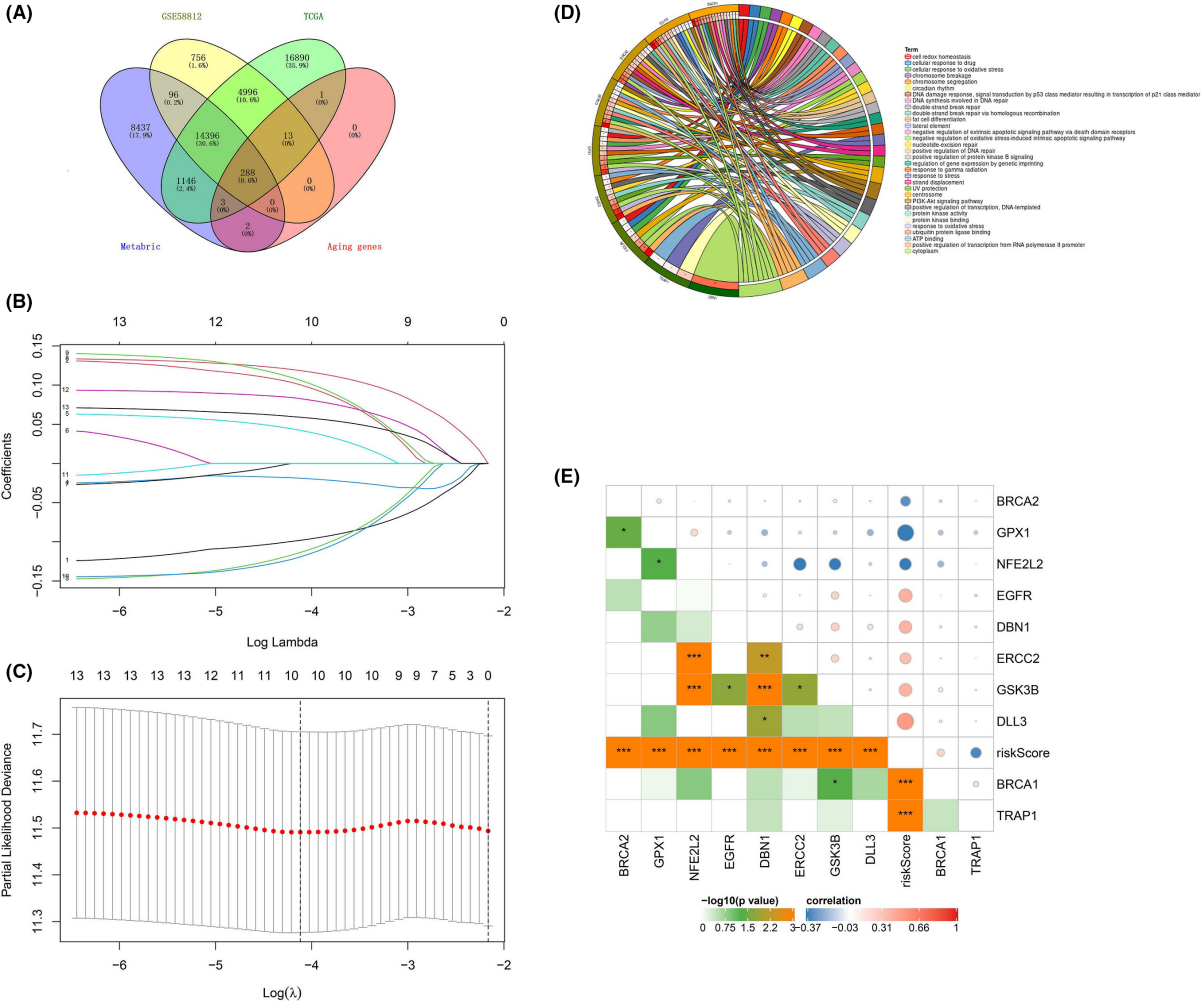
Selection of prognostic ARGs and construction of a risk model. (A) Venn diagram depicts 288 aging‐related genes in the three cohorts; (B, C) The prognostic signature constructed by the minimum criterion of LASSO Cox regression algorithm; (D) The chordal graph depicts the genome function of 10 prognostic ARGs. (E) Correlation network between the 10 ARGs and risk score

**FIGURE 2:**
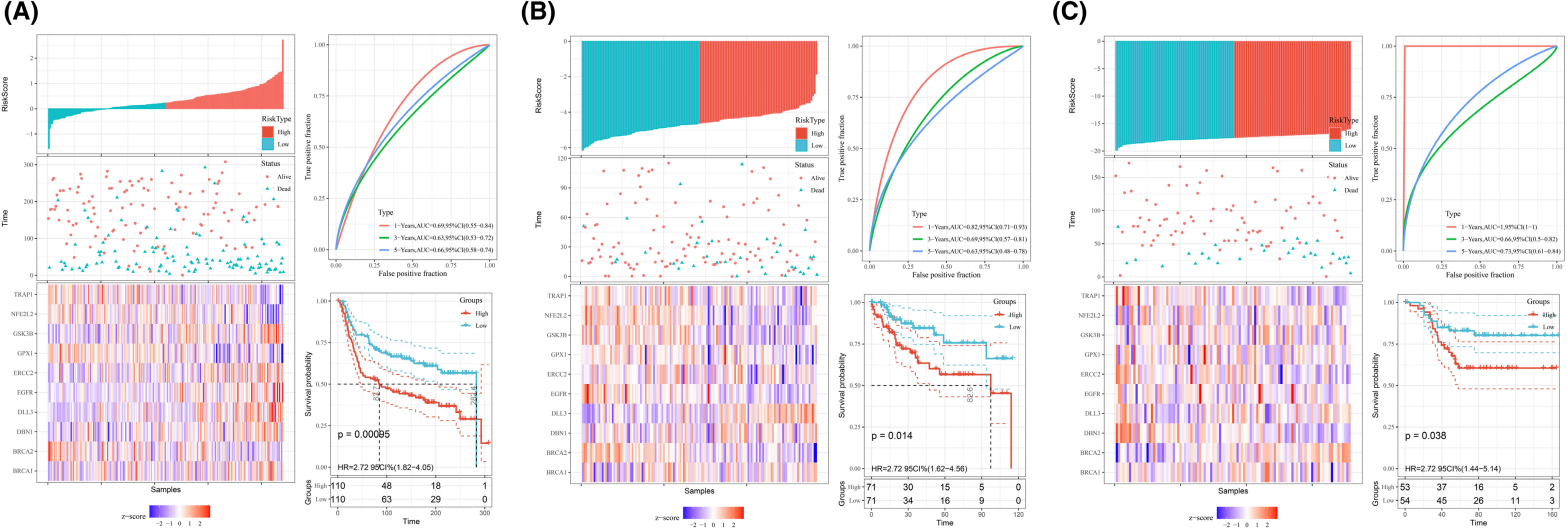
The distribution of risk score, survival analysis, and the expression level of the 10 ARGs in METABRIC cohort (A), TCGA cohort (B), and GSE58812 (C)

A total of 307 human ARGs obtained from the HAGR and 288 cross‐cohort ARGs were extracted in the three datasets (Figure 1A). Based on the available 288 ARGs, 13 ARGs were significantly correlated with OS in the univariate Cox regression analysis, and 10 prognostic ARGs were identified by performing LASSO regression model (Figure 1B,C) in METABRIC dataset. The chordal graph of genome function of these 10 ARGs was depicted in Figure 1D. Then, individual risk score was calculated based on the selected ARGs in all cohorts. Furthermore, a risk model was constructed and patients were clustered into low‐ and high‐risk groups based on their risk scores. Additionally, the correlation between ARGs and risk score were analyzed using Pearson's correlation test, which showed in Figure 1E. The distribution of risk score, survival analysis and the expression level of the 10 ARGs in all cohorts are shown in Figure 2A–C.

### 3.2 Functional enrichment analysis

**FIGURE 3:**
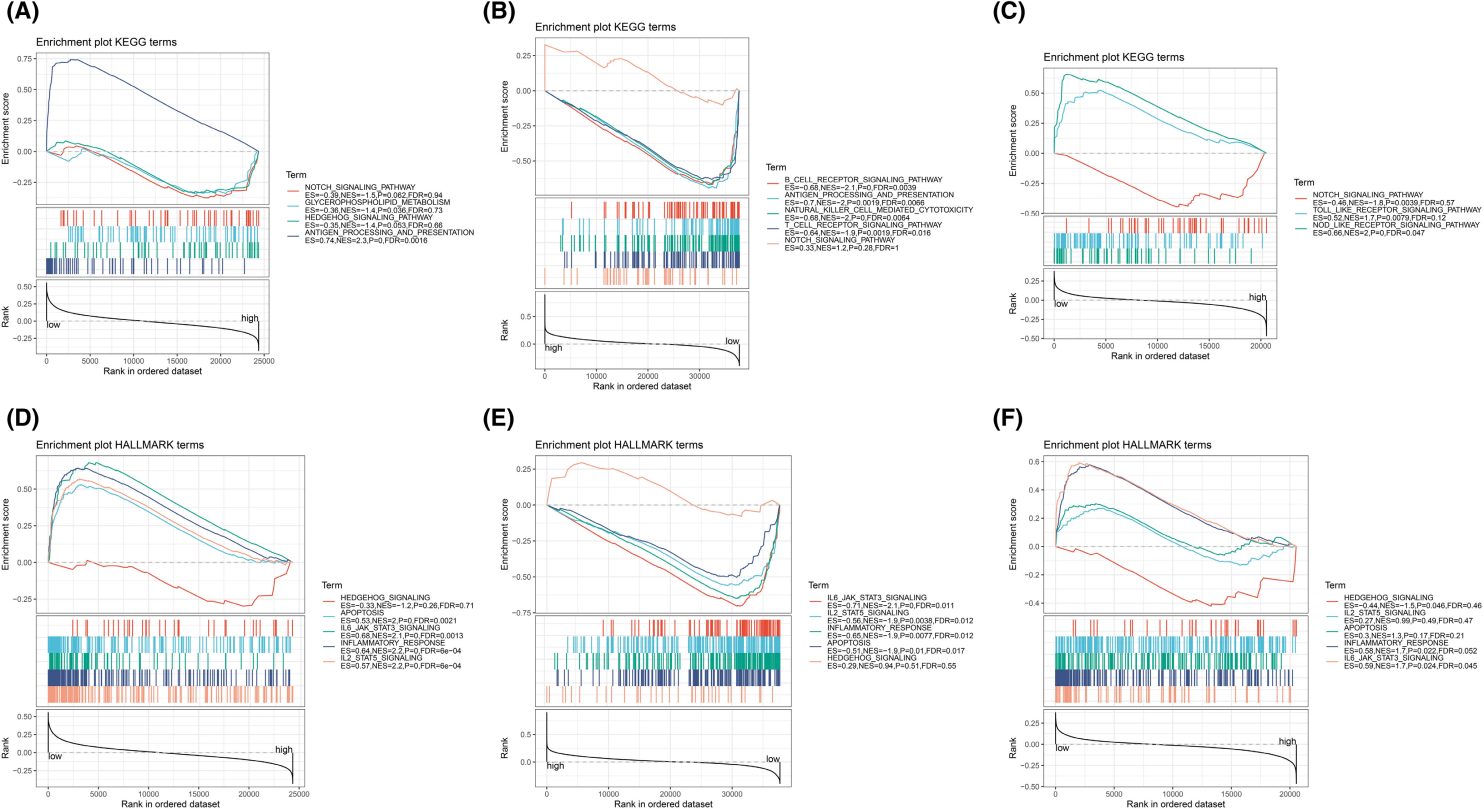
Functional enrichment analysis of the risk model. GSEA shows the key differential signaling pathways between the low‐ and high‐risk group in METABRIC cohort (A), TCGA cohort (B), and GSE58812 (C); GSEA shows the key differential cancer hallmark between the low‐ and high‐risk group in METABRIC cohort (D), TCGA cohort, (E) and GSE58812 (F)

GSEA revealed that Notch signaling pathway was significantly enriched in the high‐risk group (Figure 3A–C); In terms of cancer hallmark, apoptosis, IL2‐STA5 signaling, and IL6‐JAK‐STAT3 signaling were the most relevant cancer hallmark in the low‐risk group, while hedgehog signaling pathway was dramatically enriched in the high‐risk group (Figure 3D–F). Pertaining to gene annotation (GO) analysis, regulation of immune response was the most relevant biological process (BP), secretory granule membrane was the most relevant cellular component (CC), and chemokine binding was the most relevant molecular function (MF) in the low‐risk group (Figure S1).

### 3.3 Estimation of the tumor immune microenvironment

**FIGURE 4:**
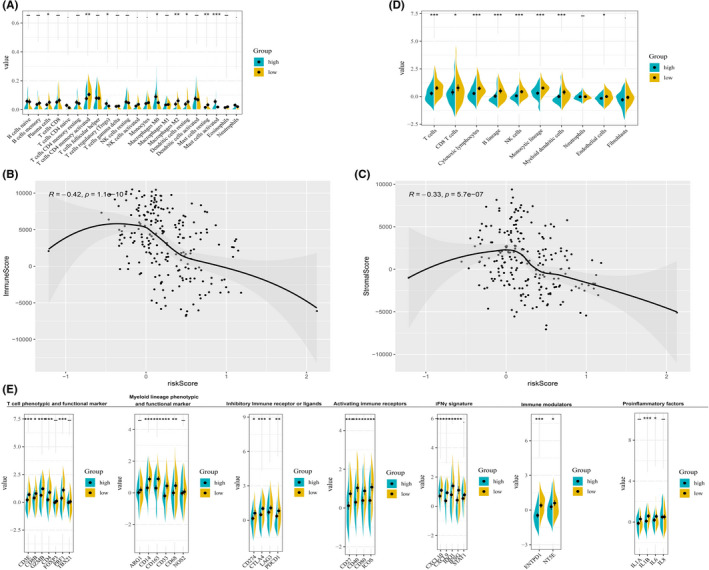
Association of tumor immune profiles with the risk model. CIBERSORT analyses between the low‐ and high‐risk groups in METABRIC cohort (A); ESTIMATE analyses between the low‐ and high‐risk groups in METABRIC cohort (B,C); MCP‐counter analyses between the low‐ and high‐risk groups in METABRIC cohort (D). The expression of immune profiles and proinflammatory factors between the low‐ and high‐risk groups in METABRIC cohort (E)

CIBERSORT algorithm revealed that patients in the low‐risk group were characterized by more antitumor immune cells (plasma cells, activated memory CD4 + T cells, and activated mast cells), while regulatory T cells and M0 macrophages were significantly enriched in patients with high‐risk scores (Figure 4A and Figure S2A, S3A). ESTIMATE algorithm confirmed that crucially negative association of the risk score with the immune score and the stromal score (Figure 4B,C and Figure S2B,C, S3B,C). MCP‐counter indicated that the low‐risk subtype was correlated with a higher level of activated immune cells (B cells, CD8+ T cells, NK cells, and cytotoxic lymphocytes) (Figure 4D and Figure S2D, S3D).

### 3.4 Association of the expression of proinflammatory factors and immune profiles with the risk model

Increasing evidence showed that chronic inflammation plays a vital role in the aging process of immune cells. We then explored the expression level of proinflammatory factors in different risk groups. As shown in Figure 4E and Figure S2E, S3E, IL‐1A, IL‐1B, IL‐8, and IL‐18 were significantly higher expressed in the low‐risk groups than those in the high‐risk groups. Besides, immune‐related signatures were prominent differentially expressed between the low‐ and high‐risk groups (Figure 4E and Figure S2E, S3E). Activating immune profiles, like the phenotypic and functional marker of T cells (CD3E, CD4, CD8B, GZMB, and PRF1), IFNγ signature (CXCL9, CXCL10, IDO1, and IFNG), activating immune receptors (CD27, CD40, CD80, and ICOS), and immune checkpoint markers (CTLA4, CD274, LAG3, and PDCD1) were observed in the low‐risk group, which indicated that the risk model was able to serve as an effective indicator for Immunotherapeutic response.

### 3.5 Identification of novel candidate compounds targeting the selected ARGs

**FIGURE 5:**
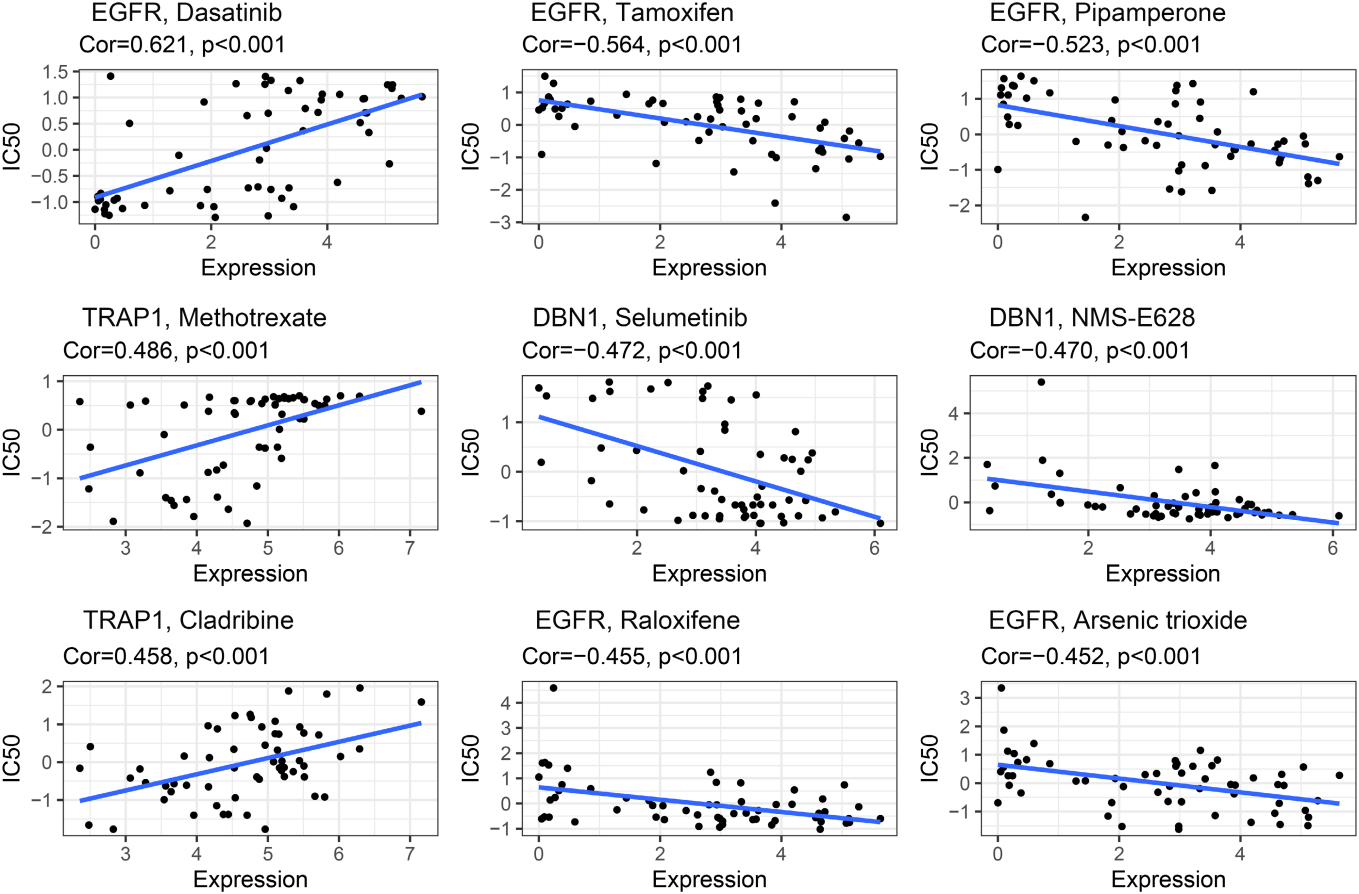
Identification of novel candidate compounds targeting the selected ARGs

To explore potential compounds targeting the selected ARGs for the treatment of TNBC, we calculated therapeutic response based on the inhibitory centration (IC50) value of multidrug available in the CellMiner database. The result showed that the IC 50 of Dasatinib appeared to be negatively correlated with EGFR, while the IC 50 of tamoxifen, pipamperone, raloxifene, and arsenic trioxide were positively associated with EGFR. Similarly, a negative correlation was observed between the IC 50 of Selumetinib and NMS‐E628 with DBN1, while a significant positive correlation was observed between the IC 50 of methotrexate and cladribine with TRAP1 (all *p* < 0.001) (Figure 5). The above finding suggested that the risk model we constructed might serve as a chemosensitivity predictor for TNBC patients.

### 3.6 Overall gene mutation and tumor mutation burden analysis

**FIGURE 6:**
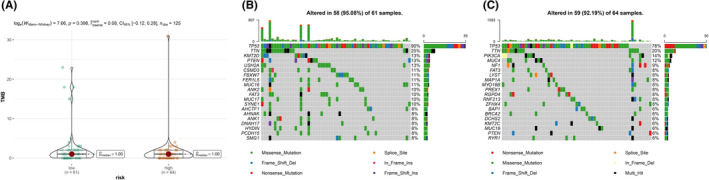
Tumor mutation burden and overall genes mutation analysis based on the risk model. The difference of tumor mutational burden between the low‐ and high‐risk groups in TCGA cohort (A); Waterfall plots depict the top 20 mutated genes in the low‐ (B) and high‐risk groups in TCGA cohort (C)

Then, we estimated the TMB based on tumor‐specific mutated genes in TCGA dataset. The result showed that although a distinct immune response was observed in different risk groups, the TMB had no significant diversity between the high‐ and the low‐risk group (Figure 6A). Using the R package maftools, we next investigated the overall gene mutation in different risk groups. As shown in Figure 6B,C, TP53 and TTN were the driver genes with the highest alteration frequency both in the high‐ and low‐risk group, and dramatical gene mutation diversity were observed between the high‐ and low‐risk groups. The result indicated the risk model might exert an effect on the genomic heterogeneity of TNBC.

### 3.7 Construction and evaluation of the ARGs‐based nomogram

**FIGURE 7:**
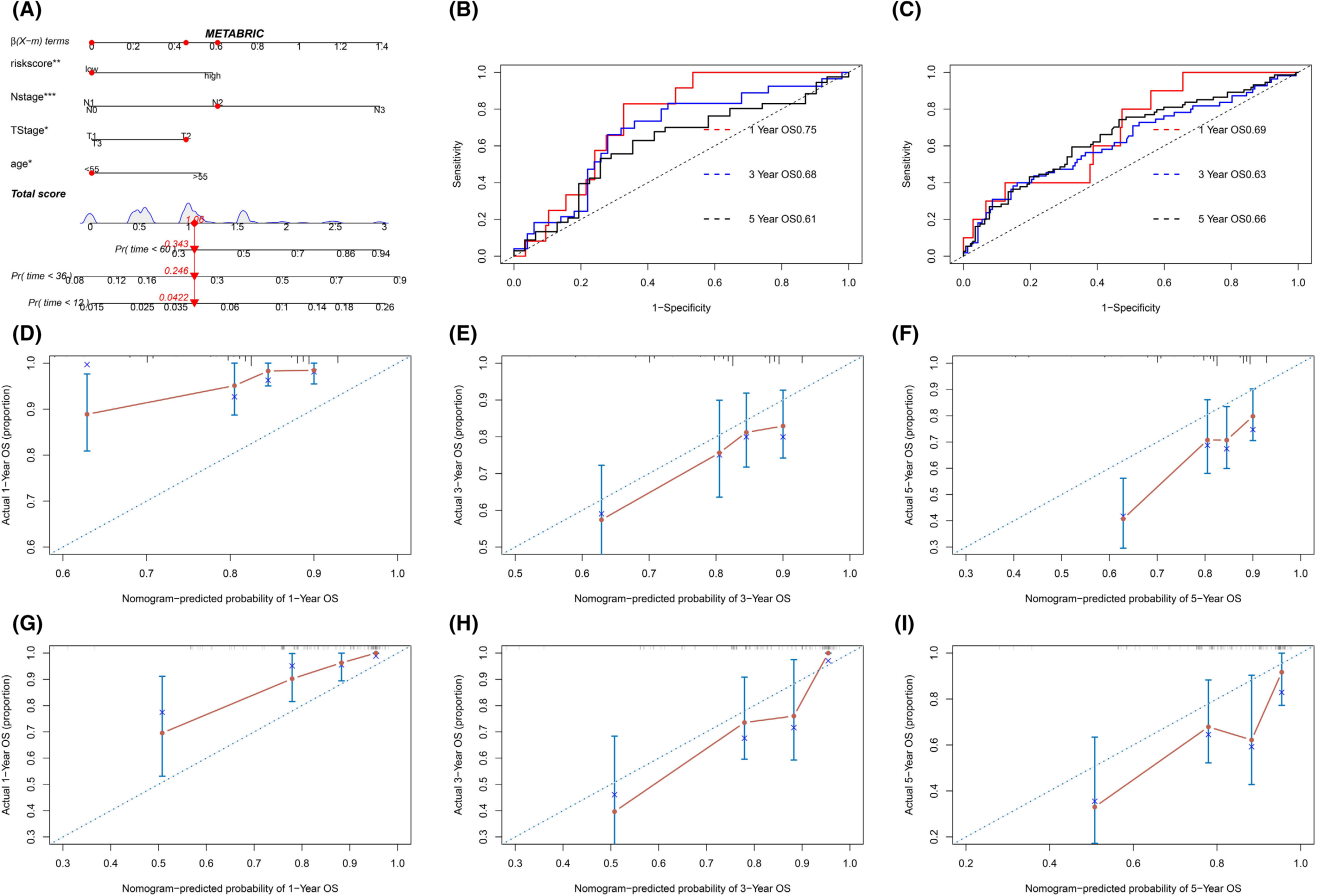
Construction and evaluation of the ARGs based nomogram. (A) A nomogram based on age, tumor stage, node stage, and the risk model were constructed; time‐ROC curves measuring the predictive value of the risk model in METABRIC (H) and TCGA (I) cohorts; Calibration plots of the nomogram for predicting the probability of OS at 1, 3, and 5 years in METABRIC (D‐F) and TCGA (G‐I) cohorts

Subsequently, we established a nomogram comprising the independent prognostic factors (age, tumor stage, node stage, and the risk model) in METABRIC cohort (Figure 7A). The AUC value showed moderate predictive accuracy in predicting 1‐, 3‐, and 5‐year OS in the training (METABRIC) and testing (TCGA) sets (Figure 7B,C). Furthermore, the calibration plots showed that reliable consistency between the actual and nomogram predicted probability of 1‐, 3‐, and 5‐year OS in the training (METABRIC) (Figure 7D–F) and testing (TCGA) sets (Figure 7G–I).

### 3.8 The mRNA levels and prognostic value of 10 selected ARGs in our cohort

**FIGURE 8:**
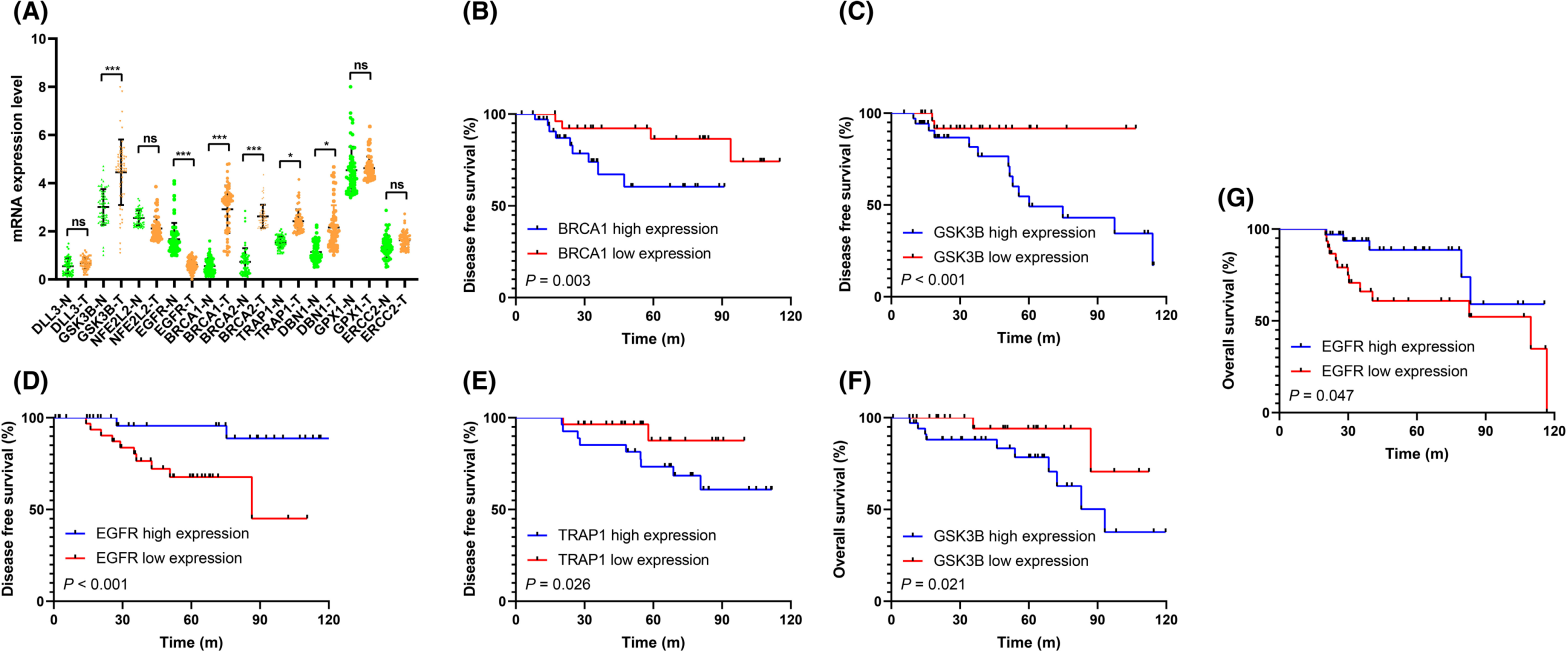
The mRNA levels and prognostic value of 10 selected ARGs in our cohort. (A) Comparison of mRNA expression levels of 10 selected ARGs in adjacent normal tissues (N) and TNBC (T) tissues by RT‐ qPCR assay. Kaplan–Meier curve shows the survival diversity between different expression of BRCA1 (B), GSK3B (C,F), TRAP1 (E), and EGFR (D,G) in our cohort. Non‐significant (ns) *p* > 0.05, *p < 0.05, **p < 0.01, and ***p < 0.001

The RT‐qPCR assay showed that the mRNA expression level of 10 selected ARGs (BRCA1, BRCA2, DLL3, DBN1, GSK3B, GPX1, TRAP1, ERCC2, EGFR, and NFE2L2) in adjuvant tumor tissue and TNBC tissues. In detail, EGFR mRNA was downregulated, while BRCA1, BRCA2, GSK3B, TRAP1, and DBN1 were significantly upregulated in TNBC samples compared with that in the paired ANTs (Figure 8A). Furthermore, Kaplan–Meier survival analysis showed that high expression of BRCA1 and TRAP1 were significantly associated with worse DFS (Figure 8B,E). GSK3B high expression (Figure 8C,F) and EGFR down expression (Figure 8D,G) were correlated with worse DFS and OS in TNBC, which was in accord with the bioinformatics results.

## 4 DISCUSSION

Increasing evidence suggested that cellular senescence plays a vital role in the pathogenesis and development of multiple tumor types. Previous studies found that aging‐tumor cells can restore proliferative activity and metabolic activity to promote tumor recurrence and metastasis.^29^ Furthermore, SASP secreted by senescent cells contains pro‐cancer inflammatory factors that evade immune surveillance, thus promoting tumor initiation and development.^40^ Besides, the adaptive immune system remodeled by the aging process would affect the response to immune checkpoint blockade.^41^ In this background, some aging‐related gene signatures for prognosis and immune response have been analyzed in different cancer types, like colorectal cancer, glioma, and head and neck squamous cell carcinoma.^42^, ^43^, ^44^, ^45^ However, aging‐related pathologic processes in the development and malignancy of TNBC are still unclear. Exploring the molecular function and mechanism of ARGs in TNBC is important for identifying the role of age‐dependent changes in the formation and malignancy of TNBC.

In this study, we performed a systematic bioinformatics analysis to establish and substantiate an aging‐related gene signature to predict prognosis, immune response, TMB, and therapeutic response in TNBC. A risk model based on 10 selected ARGs was then established, which served as an independent risk factor for predicting the prognosis of TNBC both in the training (METABRIC) and validation (TCGA) datasets. Moreover, the nomogram we constructed had a favorable predictive performance for OS in TNBC patients.

Next, we explored the mRNA levels and prognostic value of 10 selected ARGs in our cohort by RT‐qPCR assay. In accord with the bioinformatics results, we found most of the selected ARGs were differentially expressed in TNBC samples. Kaplan–Meier curve further indicated that differential expression of these selected ARGs were significantly associated with clinical outcomes in TNBC patients.

GSEA showed that Notch signaling pathway and hedgehog signaling pathway were significantly enriched in the high‐risk group; while the low‐risk group was markedly enriched in apoptosis, IL2‐STAT5, and IL6‐JAK‐STAT3 signaling pathways. Other biological features, like regulation of immune response, was found enriched in the low‐risk group. It is well established that Notch and hedgehog signaling pathways are involved in cancer stem cell proliferation, metastasis, sphere‐forming capacity, and chemotherapeutic sensitiveness in TNBC.^46^, ^47^, ^48^, ^49^ Xie et al. reported that IL6/JAK/STAT3 signaling pathway was involved in the progression of invasion and migration induced by Ilamycin C in TNBC.^50^ Considering that, the risk model based on the 10 ARGs and their relative biological function and signaling pathways may involve in the development and malignancy of TNBC.

For the relationship of the ARGs‐based risk model and immune profiles, CIBERSORT algorithm showed that patients with high‐risk scores had a remarkably higher percentage of immune‐suppressors, like regulatory T cells and M0 macrophages, while immune‐effective cells were enriched in the low‐risk group. Besides, ESTIMATE algorithm revealed that both immune and stromal score were negatively associated with the risk score. Furthermore, MCP‐counter indicated that activated immune cells were sharply decreased in the high‐risk group. These results indicated heterogeneous immune status within the different risk groups.

Previous studies demonstrated that the proinflammatory factors of SASP play a vital role in the development of inflammation and tumor immunosuppression.^24^, ^51^ In this study, we substantiated a crucially positive correlation of the expression of proinflammatory factors with our ARGs‐based risk model in TNBCs. Furthermore, amounting evidence indicated that aging is associated with immune microenvironment known as “immunosenescence” that could impact the efficacy and safety profile of immune checkpoint inhibitors.^41^, ^52^, ^53^ Here, we found a lower level of immune checkpoint markers in the high‐risk group, which meant the therapeutic response to immune checkpoint inhibitors may be affected by the aging status in TNBC.

Furthermore, we estimated the TMB and overall genes mutation in different risk groups. The clinical trial (GeparNuevo) confirmed that TMB could be an independent PCR predictor for neoadjuvant immune checkpoint inhibitors in TNBC.^54^ Another research conducted by Gao et al. revealed that the characteristics of TMB were associated with prognosis and immunotherapeutic response in TNBC.^55^ Results in our study showed that the TMB had no significant diversity between different risk groups. While dramatical gene mutation diversity was observed between differential risk groups, which indicated the ARGs‐based risk model might exert an effect on the genomic heterogeneity of TNBC.

Next, we calculated the therapeutic response of various molecular based on the selected ARGs, a negative association was observed between the IC 50 of dasatinib with EGFR, and the IC 50 of selumetinib, and NMS‐E628 with DBN1; While a positive correlation was observed between the IC 50 of tamoxifen, pipamperone, raloxifene, and arsenic trioxide with EGFR, and the IC 50 of methotrexate and cladribine with TRAP1 (all *p* < 0.001); which indicated our risk model may serve as a potential indicator for chemotherapeutic response in TNBC patients.

Among the ARGs in the risk model, BRCA1 and BRCA2 have been confirmed occurred mutation in TNBC, and were correlated with higher tumor grade and earlier age at menarche.^56^ Moreover, some researches demonstrated that PARP inhibitors combined with immune checkpoint blockade might be a potential therapeutic strategy for BRCA1‐mutated TNBC.^57^ For EGFR, which is also known as a gene with hot spot mutation in multiple tumor types. Amounting studies confirmed that the expression of EGFR was associated with poor survival in TNBC,^58^ inhibiting the expression of EGFR effectively blocking cancer stem cell clustering and lung metastasis of TNBC.^59^ Similarly, Eunkyung et al. found that GPX1 induced the malignant and metastasis of TNBC cells by interacting with FAK kinase.^60^ Qin et al. observed that NFE2L2, could be a prominent regulator of cellular antioxidant response by sensitizing cancer stem cells in TNBC.^61^ These studies provided a novel biological function and therapeutic strategy of these ARGs in TNBC.

In this study, we developed and validated a risk model based on 10 ARGs to predict prognosis, immune response, TMB, and therapeutic response in TNBC patients. Results showed that the risk model was an independent factor for survival in TNBC, and it might serve as an effective indicator for immunotherapeutic and chemotherapeutic response in TNBCs. Nevertheless, several limitations in this study should be noted. First, the results were analyzed and verified based on the METABRIC, TCGA, and GSE58812 datasets. External verification are needed based on our own data in the future. Second, further experimental studies are warranted to elucidate the underlying biological function and mechanism of these ARGs in TNBC.

In conclusion, we developed and validated aging‐related genes‐based risk model, which may serve as a potential biomarker for prognosis and individualized treatment in TNBC patients.

## CONFLICT OF INTEREST

No conflict of interest needed to be declared.

## AUTHOR CONTRIBUTIONS

Conception and design by Xia Yang, analyzing and processing data by Xia Yang, Writing and revising by Xia Yang and Yanhua Sun, Supervising by Xia Liu and Zhinong Jiang. All authors approved the final submitted manuscript.

## ETHICS APPROVAL AND CONSENT TO PARTICIPATE

The study was approved by Ethics Institutional Review Board of first affiliated hospital of Shenzhen University. Written informed consent was obtained from all patients of the study. The patients' records/information were anonymized and de‐identified prior to analysis.

## CONSENT FOR PUBLICATION

All authors approved for publication.

## Supporting information


Figure S1

Figure S2

Figure S3

Table S1

## Data Availability

Data sharing is not applicable to this article as no new data were created or analyzed in this study.
