# Supplementary figures and images for: A risk model of 10 aging‐related genes for predicting survival and immune response in triple‐negative breast cancer
Source: Cancer Med. 2022 Mar 16;11(16):3182–93. doi: 10.1002/cam4.4674 (PMC9385588; doi:10.1002/cam4.4674)

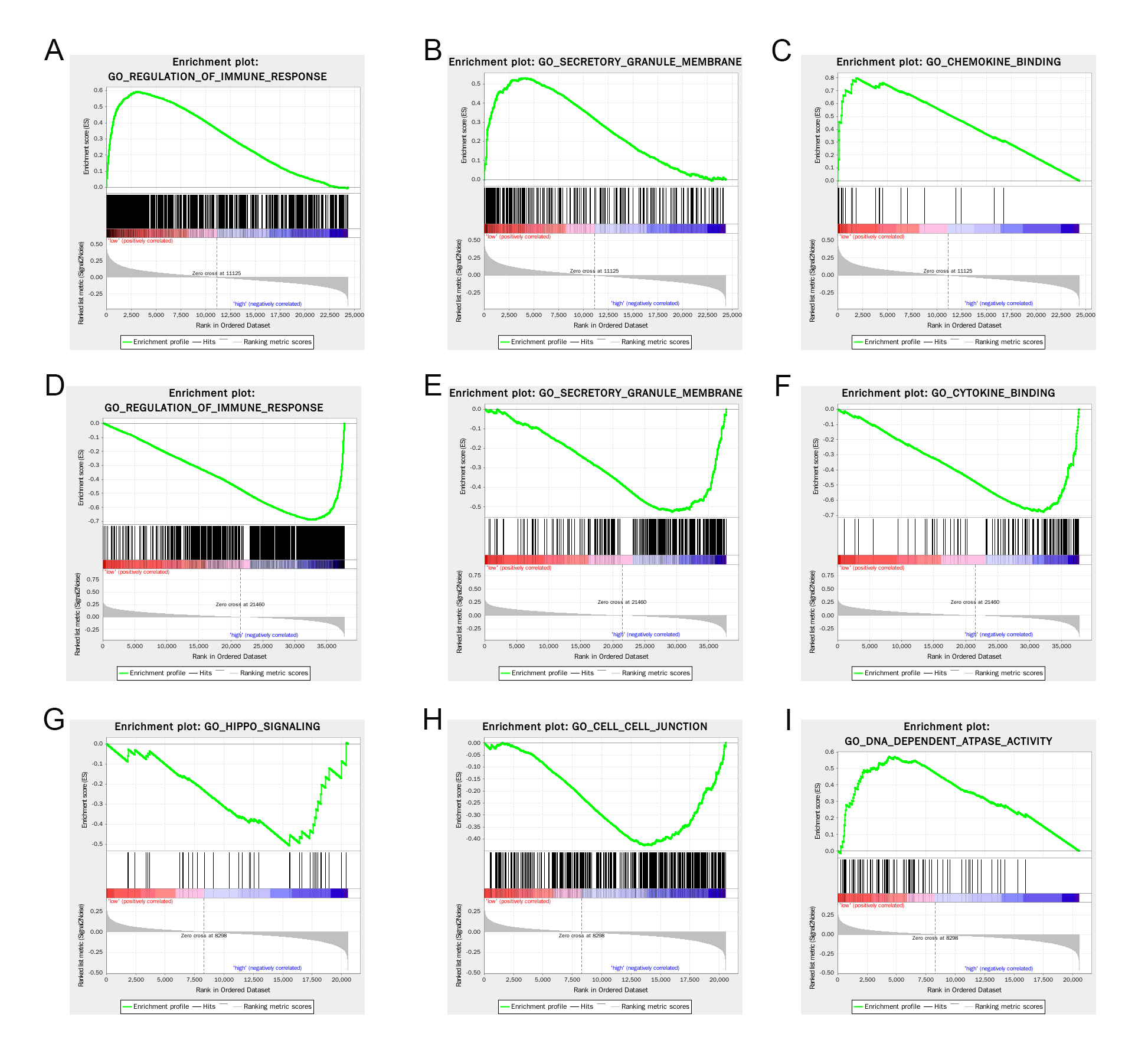

Supplement: Supplementary file 1 — Figure S1 [file CAM4-11-3182-s002.tif]

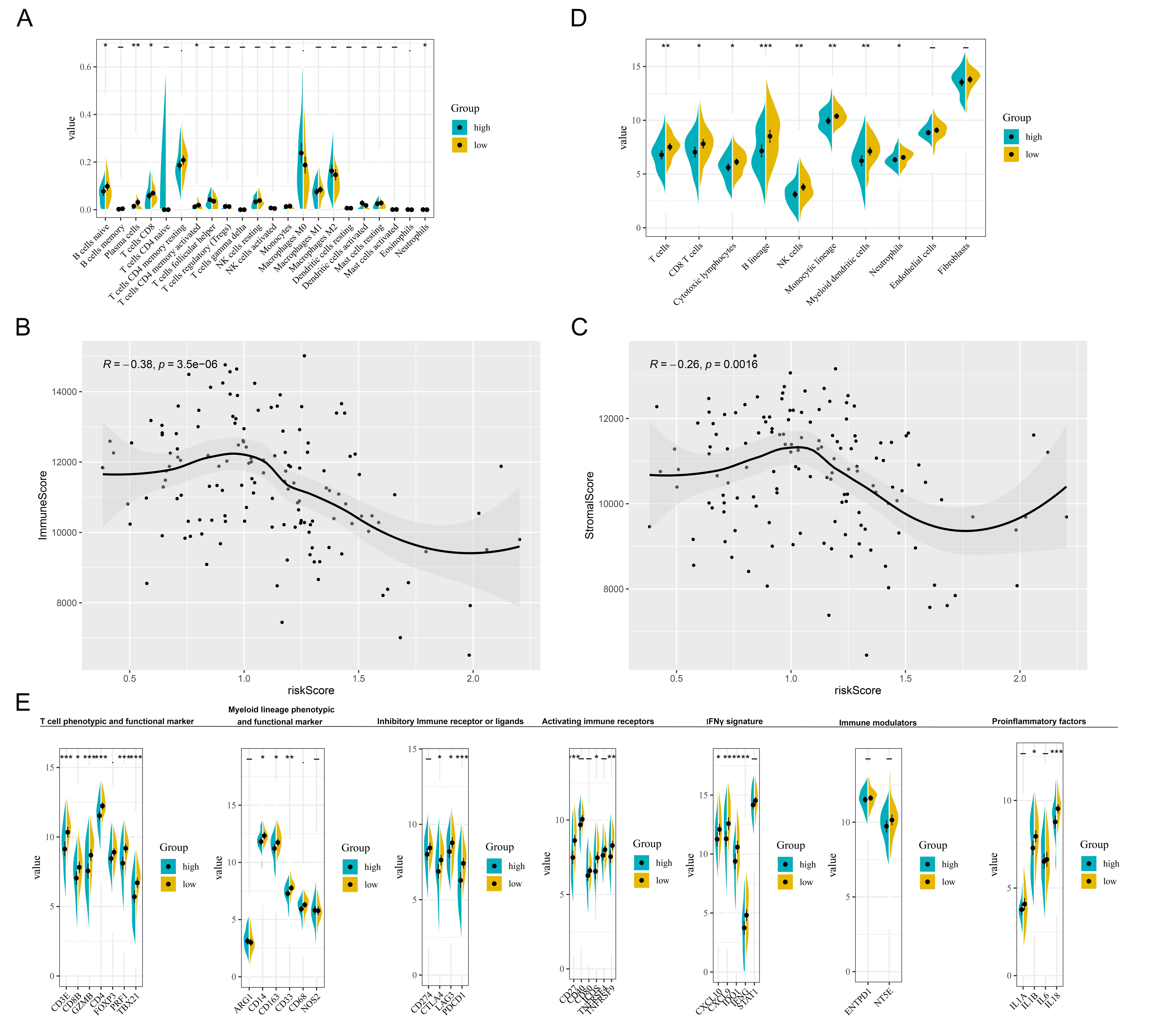

Supplement: Supplementary file 2 — Figure S2 [file CAM4-11-3182-s001.jpg]

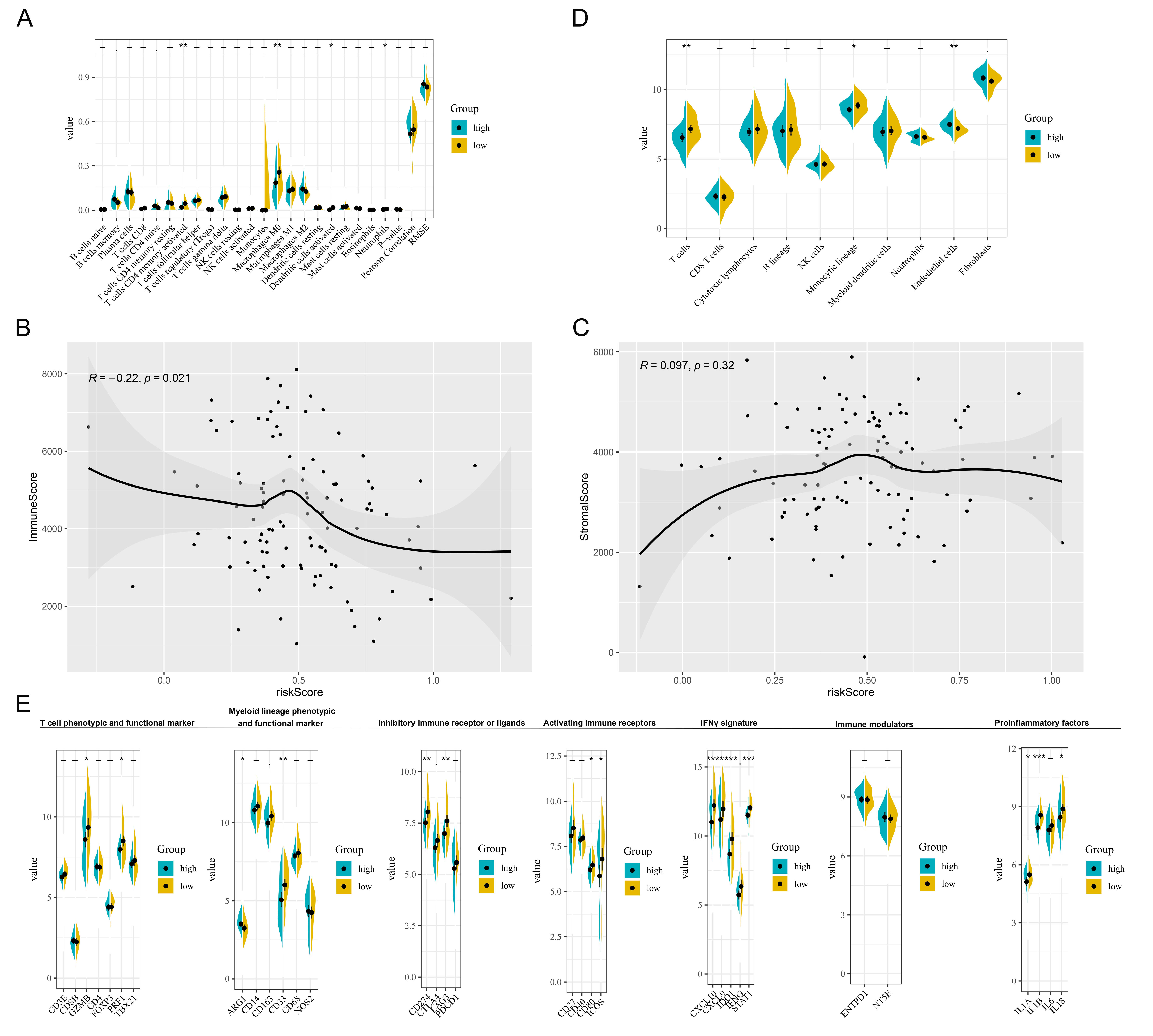

Supplement: Supplementary file 3 — Figure S3 [file CAM4-11-3182-s003.jpg]
