# Supplementary material for: A risk model of 10 aging‐related genes for predicting survival and immune response in triple‐negative breast cancer
Source: Cancer Med. 2022 Mar 16;11(16):3182–93. doi: 10.1002/cam4.4674 (PMC9385588; doi:10.1002/cam4.4674)
Supplement: Supplementary file 4 — Table S1 [file CAM4-11-3182-s004.docx]

Table S1 Primers for real-time PCR.

| Gene |  | Sequence (5' -> 3') |
| --- | --- | --- |
| BRCA1 | Forward Primer | GAAACCGTGCCAAAAGACTTC |
|  | Reverse Primer | CCAAGGTTAGAGAGTTGGACAC |
| BRCA2 | Forward Primer | CACCCACCCTTAGTTCTACTGT |
|  | Reverse Primer | CCAATGTGGTCTTTGCAGCTAT |
| EGFR | Forward Primer | AGGCACGAGTAACAAGCTCAC |
|  | Reverse Primer | ATGAGGACATAACCAGCCACC |
| ERCC2 | Forward Primer | AGAAGGTGATTGAAGAGCTTCG |
|  | Reverse Primer | ACCTCAGGGTGAATACACAAGT |
| DLL3 | Forward Primer | CGTCCGTAGATTGGAATCGCC |
|  | Reverse Primer | TCCCGAGCGTAGATGGAAGG |
| TRAP1 | Forward Primer | AGGACGACTGTTCAGCACG |
|  | Reverse Primer | CCGGGCAACAATGTCCAAAAG |
| GSK3B | Forward Primer | GGCAGCATGAAAGTTAGCAGA |
|  | Reverse Primer | GGCGACCAGTTCTCCTGAATC |
| DBN1 | Forward Primer | CAACTGGGTGGGCGAAGAT |
|  | Reverse Primer | TGCTGGCGTTCACGATCAC |
| GPX1 | Forward Primer | CAGTCGGTGTATGCCTTCTCG |
|  | Reverse Primer | GAGGGACGCCACATTCTCG |
| NFE2L2 | Forward Primer | TCAGCGACGGAAAGAGTATGA |
|  | Reverse Primer | CCACTGGTTTCTGACTGGATGT |
